# Prediction of carotid plaque by blood biochemical indices and related factors based on Fisher discriminant analysis

**DOI:** 10.1186/s12872-022-02806-3

**Published:** 2022-08-15

**Authors:** Jian Hu, Fan Su, Xia Ren, Lei Cao, Yumei Zhou, Yuhan Fu, Grace Tatenda, Mingfei Jiang, Huan Wu, Yufeng Wen

**Affiliations:** 1 School of Public Health, Wannan Medical College, 22 West Wenchang Road, Wuhu, 241002 Anhui Province People’s Republic of China; 2 School of Clinical Medicine, Wannan Medical College, 22 West Wenchang Road, Wuhu, 241002 Anhui Province People’s Republic of China; 3 School of Laboratory Medicine, Wannan Medical College, 22 West Wenchang Road, Wuhu, 241002 Anhui Province People’s Republic of China

**Keywords:** Carotid plaque, Carotid plaque location, Prediction model, Fisher discriminant analysis

## Abstract

**Objective:**

This study aims to establish the predictive model of carotid plaque formation and carotid plaque location by retrospectively analyzing the clinical data of subjects with carotid plaque formation and normal people, and to provide technical support for screening patients with carotid plaque.

**Methods:**

There were 4300 subjects in the ultrasound department of Maanshan People's Hospital collected from December 2013 to December 2018. We used demographic and biochemical data from 3700 subjects to establish predictive models for carotid plaque and its location. The leave-one-out cross-validated classification, 600 external data validation, and area under the receiver operating characteristic curve (AUC) were used to verify the accuracy, sensitivity, specificity, and application value of the model.

**Results:**

There were significant difference of age (*F* = − 34.049, *p* < 0.01), hypertension (*χ*^*2*^ = 191.067, *p* < 0.01), smoking (*χ*^*2*^ = 4.762, *p* < 0.05) and alcohol (*χ*^*2*^ = 8.306, *p* < 0.01), Body mass index (*F* = 15.322, *p* < 0.01), High-density lipoprotein (HDL) (*F* = 13.840, *p* < 0.01), Lipoprotein a (Lp a) (*F* = 52.074, *p* < 0.01), Blood Urea Nitrogen *(F* = 2.679, *p* < 0.01) among five groups. Prediction models were built: carotid plaque prediction model (Model CP); Prediction model of left carotid plaque only (Model CP Left); Prediction model of right carotid plaque only (Model CP Right). Prediction model of bilateral carotid plaque (Model CP Both). Model CP (Wilks' lambda = 0.597, *p* < 0.001, accuracy = 78.50%, sensitivity = 78.07%, specificity = 79.07%, AUC = 0.917). Model CP Left (Wilks' lambda = 0.605, *p* < 0.001, accuracy = 79.00%, sensitivity = 86.17%, specificity = 72.70%, AUC = 0.880). Model CP Right (Wilks' lambda = 0.555, *p* < 0.001, accuracy = 83.00%, sensitivity = 81.82%, specificity = 84.44%, AUC = 0.880). Model CP Both (Wilks' lambda = 0.651, *p* < 0.001, accuracy = 82.30%, sensitivity = 89.50%, specificity = 72.70%, AUC = 0.880).

**Conclusion:**

Demographic characteristics and blood biochemical indexes were used to establish the carotid plaque and its location discriminant models based on Fisher discriminant analysis (FDA), which has high application value in community screening.

**Supplementary Information:**

The online version contains supplementary material available at 10.1186/s12872-022-02806-3.

## Introduction

According to the Organization for Economic Cooperation and Development, chronic diseases account for major causes of death and health problems: 60 percent (about 35 million) of the world’s population is estimated to die from chronic disease. Currently, cardiovascular diseases (CVD) are the leading cause of morbidity and mortality around the world [1–3]. Carotid plaque in the carotid artery is the most significant risk factor for cardiovascular disease. CVD can be controlled, and earlier treatment and intervention for patients with carotid plaque can effectively reduce the mortality of CVD [4]. However, the clinical diagnosis of carotid plaque is single, expensive, and complex, and can not meet the requirements for the prevention of carotid plaque [5–7]. Therefore, appropriate screening tools are integral to the early detection and prevention of major cardiovascular events.

The Fisher discriminant analysis (FDA) is efficient at distinguishing patients from normal people. This field is growing and has the potential to provide useful insights for healthcare systems. FDA was used to predict clinical decisions made by physicians and the outcomes of clinical activity for conditions based on patient features, the model has been applied to predict breast cancer, diabetes 2, and other diseases [8–12]. The establishment of an effective discriminant model has reference significance for early identification of patients with carotid plaque formation in the population, saving social resources for chronic disease prevention and control, and formulating follow-up clinical treatment plans [13–15].

However, the diagnostic value of the current prediction model about the carotid plaque is still not high, in view of the advantages of the FDA in constructing the prediction model, in this study, we aimed to establish the predictive models of carotid plaque and the location based on FDA, so as to provide technical support for early screening and intervention.

## Research materials and methods

### Study subjects

In this study, 3700 subjects underwent carotid artery ultrasound examination in the ultrasound department of Maanshan People's Hospital from 2013 to 2018. A total of 600 subjects underwent carotid artery doppler ultrasound from the physical examination center of Maanshan People's Hospital as external data to evaluate the models. The inclusion criteria were (1) no history of serious cardiovascular disease; (2) no history of tumor; (3) no history of carotid endarterectomy. (4) written informed consent to participate in the study. Exclusion criteria were (1) data missing. (2) history of carotid artery stenting; (3) history of other major diseases. All respondents signed or their family members signed the relevant informed consent.

### Questionnaire survey

The questionnaire was developed by radiologists and epidemiologists, reviewed and revised by clinicians and radiologists. The contents of the questionnaire mainly include demographic characteristics (such as age, gender, etc.), history of diseases (such as hypertension, heart disease, diabetes mellitus type 2, etc.), and behavioral characteristics (such as smoking, alcohol, etc.). All investigators were trained uniformly prior to the study, and the survey was conducted in a face-to-face manner between the investigators and participants themselves.

### Definition

Hypertension was defined as taking antihypertensive drugs or systolic blood pressure ≥ 140 mm Hg or diastolic blood pressure ≥ 90 mm Hg [16]. Diabetes mellitus was defined as Plasma glucose concentration ≥ 11.1 mmol/L at any time; fasting plasma glucose (FPG) ≥ 7.0 mmol/L or oral glucose tolerance test (OGTT) ≥ 11.1 mmol/L for 2 h [17]. Smoking was defined as Smokers who have smoked continuously or cumulatively for 6 months or more in their life [18]. Alcohol consumption was defined as no less than 50 ml of 56 liquor per day [19].

### Body examination

Height measurement required subjects to stand barefooted on the floor of the height meter, with their trunks naturally straight, heads straight, with eyes focused in front, upper limbs drooping naturally, and legs straight. The recorded data on height were accurate to 0.1 cm. Body weight measurement required barefooted subjects to wear shorts and stand naturally in the center of the weight pedal so as to keep their bodies stable. The recorded data were accurate to 0.1 kg. The BMI was calculated by dividing body weight (kg) with the square of the height (m) i.e., m^2^, to an accuracy of 0.01 kg/m^2^.

### Clinical blood biochemical examination

Fasting venous blood (5 mL) was collected from each subject in the morning, and all samples were analyzed within 24 h. The indicators included triglyceride (TG), total cholesterol (TC), glucose (GLU), low-density lipoprotein (LDL) and high-density lipoprotein (HDL), blood urea nitrogen (BUN), Uric Acid (UA), Apolipoprotein a (APO a), Apolipoprotein b (APO b), creatinine (Cr), cystatin C (CysC) were measured using the Hitachi 7600 automatic biochemical analyzer.

### Carotid plaque detection

**Fig. 1:**
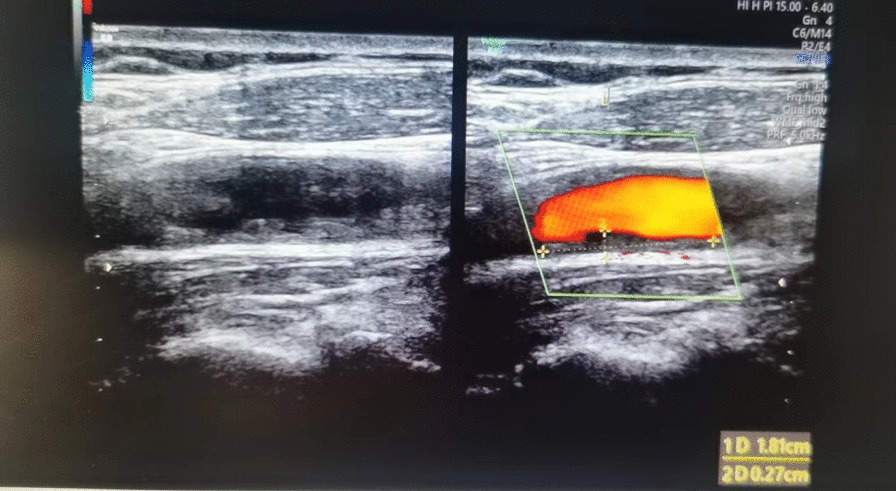
measurement of carotid IMT

Patients rest peacefully for 10 min before being examined to keep their heart rate and blood pressure stable. The neck was exposed fully before the examination. 5–13 MHZ linear array probes were used by a professional physician (Aloka-A7 and Aloka-A10, Tokyo; Philips-IU22, Colombia). The internal and external carotid arteries were measured parallel and forward from the left and right common carotid arteries through the bifurcation of the carotid artery. If the patients have plaque, the site, thickness and size of the plaque should also be measured (Fig. 1).

### Carotid plaque criteria

When the carotid intima-media thickness (IMT) was greater than or equal to 1.5 mm, it was judged as the carotid plaque group, and when the carotid IMT was less than 1.0 mm, it was judged as the normal group [20, 21]. All the participants were divided into five groups according to the location of carotid plaque: CP group (CP in left or right carotid artery), CP left group (CP only in left carotid artery), CP right group (CP only in right carotid artery), CP both group (CP in both sides of carotid artery) and none group (none of CP in carotid artery).

### Construction of the discriminant model

Regarding two-class problems, the FDA method identifies a projection vector to maximize between-class scatter matrix while minimizing within-class scatter matrix in the feature space [22, 23].The aim here is to find a linear function equation set.

$${\text{Y}}_{{{\text{CP}}}} = {\text{a}}_{{1}} {\text{X}}_{{1}} + {\text{a}}_{{2}} {\text{X}}_{{2}} + {\text{a}}_{{3}} {\text{X}}_{{3}} + \cdots + {\text{a}}_{{\text{n}}} {\text{X}}_{{\text{n}}} + {\text{C}}$$

$${\text{Y}}_{{{\text{NCP}}}} = {\text{b}}_{{1}} {\text{X}}_{{1}} + {\text{b}}_{{2}} {\text{X}}_{{2}} + {\text{b}}_{{3}} {\text{X}}_{{3}} + \cdots + {\text{b}}_{{\text{n}}} {\text{X}}_{{\text{n}}} + {\text{C}}$$

$${\text{W}}\left( {\text{X}} \right) = {\text{D}}\left( {{\text{X}},{\text{Y}}_{{{\text{CP}}}} } \right) - {\text{D}}\left( {{\text{X}},{\text{Y}}_{{{\text{NCP}}}} } \right)$$

a_n_ and b_n_ are standardized coefficients, and C is a constant, which has the following discrimination principles

$${\text{W}}\left( {\text{X}} \right) > 0,{\text{X}} \in {\text{Y}}_{{{\text{CP}}}}$$

$${\text{W}}\left( {\text{X}} \right) < 0,{\text{X}} \in {\text{Y}}_{{{\text{NCP}}}}$$

$${\text{W}}\left( {\text{X}} \right) = 0,{\text{undetermined}}$$

The optimal combination of discriminant variables was screened by the mahalanophanes distance stepwise method.

### Statistical analysis

SPSS 18.0 software was used for statistical analysis. continuous data were expressed as mean ± standard deviation (SD) and categorical variables as percentages. All the continuous data are normalized before fitting the FDA model. The differences between the CP group and the normal group were compared by the F test or chi-square test. The leave-one-out cross-validated classification was applied for the statistical significance of the model construction and the excellence of the model were evaluated by accuracy, sensitivity, and specificity. And the models were verified by 600 Physical examination crowd from Maanshan People's Hospital who underwent doppler ultrasound examination. A double-blind trial was used to test the model's ability to distinguish between plaque and healthy people. The FDA score was used to describe the application value of carotid plaque model based on the value of the AUC.

## Result

### General demographic characteristics and blood biochemical indexes of subjects

Univariate analysis showed that age (*F* = − 34.049, *p* < 0.001), gender (*χ*^*2*^ = 9.674, *p* < 0.022) hypertension (*χ*^*2*^ = 191.067, *p* < 0.001), smoking (*χ*^*2*^ = 4.762, *p* < 0.05) and alcohol (*χ*^*2*^ = 8.306, *p* < 0.001), BMI (F = 15.322, *p* < 0.001) was significantly different in the five groups. TC (*F* = 7.420, *p* < 0.001), TG (*F* = 7.236, *p* < 0.001), HDL (*F* = 13.840, *p* < 0.01), LDL (*F* = 5.211, *p* = 0.011), GLU (*F* = 12.679, *p* < 0.05), BUN (*F* = 12.679, *p* < 0.001) were significantly different from in the five groups (Table 1).

**Table 1:** General demographic characteristics and blood biochemical indexes of subjects

Variables	CP (n = 3146)	Left CP (n = 1253)	Right CP (n = 1109)	Both CP (n = 784)	None (n = 554)	*F/χ*^*2*^	*p*
Age	68.20 ± 8.53	60.12 ± 11.23	60.0 ± 11.07	69.41 ± 8.30	52.91 ± 9.95^abc^	51.180	< 0.001
Gender, Male (%)	1992 (53.8)	621 (49.6)	539 (47.7)	461 (58.8)	274 (49.0)	9.674	0.022
Hypertension (%)	2301 (73.3)	725 (57.9)	626 (56.4)	596 (76.0)	245 (44.2)	191.067	< 0.001
Smoking (%)	1116 (35.5)	387 (30.9)	342 (30.8)	301 (38.4)	170 (30.7)	4.762	< 0.05
Alcohol (%)	933 (29.7)	332 (26.5)	276 (24.9)	242 (30.9)	131 (23.6)	8.306	< 0.05
Diabetes mellitus	1360 (43.3)	295 (23.5)	219 (19.7)	349 (44.6)	258 (46.2)	5.756	0.124
BMI	23.63 ± 3.31	24.10 ± 3.44	24.17 ± 3.43	23.64 ± 3.31	24.73 ± 3.48^abc^	15.322	< 0.001
TC	4.21 ± 1.07	4.31 ± 1.05	4.33 ± 1.05	4.21 ± 1.01	4.46 ± 1.06^abc^	7.420	< 0.001
TG	1.56 ± 1.17	1.71 ± 1.32	1.68 ± 1.34	1.55 ± 1.22	1.83 ± 1.54^abc^	7.236	< 0.001
HDL	1.15 ± 0.27	1.19 ± 0.30	1.20 ± 0.30	1.15 ± 0.27	1.24 ± 0.32^abc^	13.840	< 0.001
LDL	2.33 ± 0.85	2.40 ± 0.93	2.39 ± 0.73	2.34 ± 0.79	2.48 ± 0.70^abc^	5.211	0.011
APO a	1.21 ± 0.18	1.25 ± 0.24	1.25 ± 0.23^c^	1.20 ± 0.18^b^	1.28 ± 0.27^abc^	20.511	< 0.001
APO b	0.88 ± 0.27	0.88 ± 0.26	0.88 ± 0.24	0.88 ± 0.27	0.89 ± 0.22	0.769	0.510
Lp (a)	210.50 ± 135.04	202.00 ± 135.60^c^	197.75 ± 102.31^c^	217.31 ± 137.87^ab^	138.94 ± 102.31^abc^	52.074	< 0.001
GLU	6.82 ± 3.27	7.07 ± 3.85	6.88 ± 2.90^c^	6.87 ± 3.06^b^	7.25 ± 3.29^bc^	12.679	0.007
Cr	82.32 ± 44.58	77.68 ± 23.05^c^	79.40 ± 43.66^c^	84.86 ± 50.28^ab^	76.01 ± 54.39^c^	7.959	< 0.001
BUN	6.07 ± 2.39	5.73 ± 1.73^c^	5.80 ± 1.93^c^	6.24 ± 2.75^ab^	5.72 ± 2.02^c^	12.679	0.001
CysC	1.22 ± 0.60	1.14 ± 0.26	1.18 ± 0.45	1.27 ± 0.72^ab^	1.02 ± 0.46^abc^	29.091	< 0.001
UA	324.63 ± 82.460	320.90 ± 78.71	313.35 ± 86.85^c^	329.27 ± 82.15^ab^	300.45 ± 83.98^abc^	19.490	< 0.001

The letter a indicates comparison with the left group, *p* < 0.05; the letter b indicates comparison with right group, *p* < 0.05; the letter c indicates comparison with both group; *p* < 0.05

### Basic information about external data

There were significant difference of hypertension (*χ*^*2*^ = 32.120, *p* < 0.001), smoking (*χ*^*2*^ = 4.356, *p* < 0.05) and alcohol (*χ*^*2*^ = 7.332, *p* < 0.05), creatinine (Cr) (*F* = 13.365, *p* < 0.001), Uric Acid (UA) (*F* = 31.419, *p* < 0.001), Blood Urea Nitrogen (BUN) (*F* = 65.296, *p* < 0.001) among four groups (Additional file 1: supplementary Table 1).

### Collinearity diagnosis of blood biochemical indexes

TG (t = − 4.04, *p* < 0.001, VIF = 1.636), Lp (a) (t = 10.795, *p* < 0.001,VIF = 1.073), Cr (t = − 6.32, *p* < 0.001,VIF = 3.611), Cysc (t = 7.361, *p* < 0.001, VIF = 3.281), UA (t = 4.129, *p* < 0.001, VIF = 3.611). VIF is less than 5, and there is no multicollinearity in all blood biochemical indexes, so it can be used in the following discriminant analysis (Additional file 2: supplementary Table 2).

### Establishment of Fisher discriminant equation set

In model CP, Wilks' lambda = 0.597, *χ*^*2*^ = 1196.980, *p* < 0.001. In model CP left group, Wilks' lambda = 0.605, *χ*^*2*^ = 441.594, *p* < 0.001, In model CP right group, Wilks' lambda = 0.555, *χ*^*2*^ = 479.086, *p* < 0.001. In model CP Both group, Wilks' lambda = 0.651, *χ*^*2*^ = 1586.68, *p* < 0.001. According to Wilks' lambda test, the included variables of the four models are statistically significant, and the included variables can help the model carry out correct classification and improve the accuracy of the model (Table 2).

**Table 2:** Coefficients of model equations

Variables	Model CP		Model CP Left		Model CP Right		Model CP Both	
	CP	NG	CP	NG	CP	NG	CP	NG
Constant	− 95.229	− 89.983	− 94.136	− 91.373	− 75.579	− 73.109	− 91.922	− 85.678
Age	0.871	0.659	0.708	0.552	0.646	0.477	0.954	0.722
Hypertension	10.826	11.786	11.050	11.977	10.329	11.301	9.701	11.015
Alcohol	6.349	7.315	9.197	9.646			5.294	6.048
Smoking					4.536	5.016	0.696	1.278
BMI	2.454	2.602	2.698	2.852	2.401	2.544	2.675	2.806
HDL	− 3.294	− 1.045	15.694	17.284	9.864	10.963	16.575	18.353
LDL					3.741	4.113		
Lp (a)	0.016	0.010	0.015	0.009	0.01	0.004	0.018	0.012
GLU			0.905	0.853			1.170	1.113
BUN	0.485	0.535	0.479	0.643	1.087	1.198		
UA			0.050	0.047				
APO a	36.551	35.586						

*CP* carotid plaque, *NG* normal group

### Evaluation of model quality

**Fig. 2:**
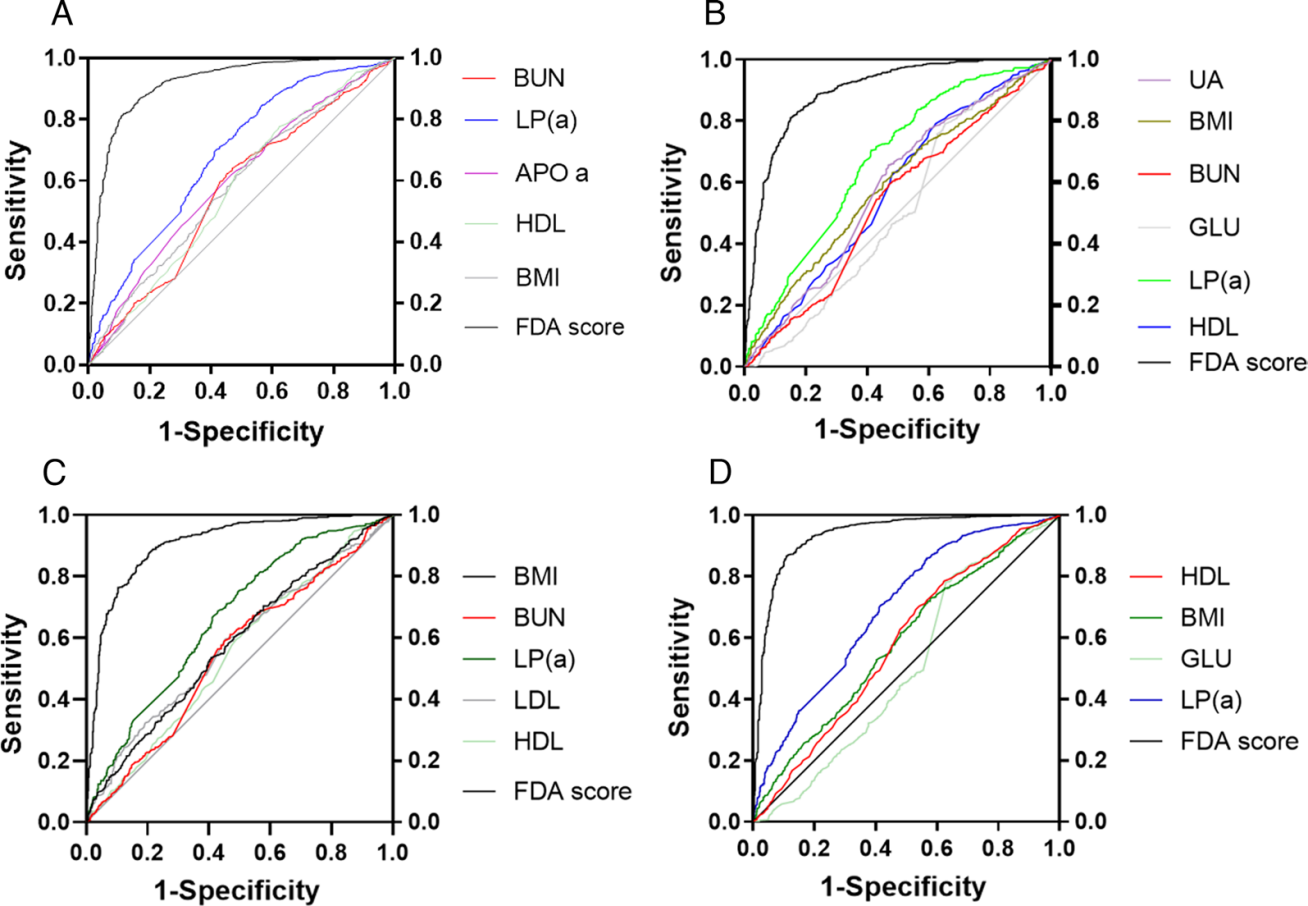
ROC curve was used to evaluate the quality of the model. **A** Model CP, FDA is better than that of the single continuous variable (BUN, LP a, HDL, BMI, Apo a). **B** Model CP Left, FDA is better than that of the single continuous variable (UA, BMI, BUN, GLU, LP a, HDL). **C** Model CP Right, FDA is better than that of the single continuous variable (BMI, BUN, LP a, HDL, LDL).**D** Model CP Both, FDA is better than that of the single continuous variable (HDL, BMI, GLU, LP a)

An important measure of the accuracy of a prediction model is the receiver operator characteristic curve (ROC curve). The AUC of the single continuous variables were all between 0.3 and 0.6 (Fig. 2), which indicated low diagnostic values. The AUC of the FDA scores was higher than the values of single continuous variables. The accuracy, sensitivity, specificity, and AUC of the model CP were 86.86%, 88.00%, 80.50%, and 0.917 (95%CI 0.903–0.931) (Additional file 3: supplementary Table 3) respectively. The accuracy, sensitivity, specificity and AUC of the model CP left were 82.90%, 83.43%, 79.96%, and 0.880 (95%CI 0.878–0.914) (Additional file 4: supplementary Table 4) respectively. The accuracy, sensitivity, specificity, and AUC of model CP right were 82.30%, 85.45%, 79.25%, and 0.905 (95%CI 0.888–0.923) (Additional file 5: supplementary Table 5) respectively. The accuracy, sensitivity, specificity, and AUC of model CP both were 87.70%, 95.54%, 68.13%, and 0.905 (95%CI 0.888–0.923) (Additional file 6: supplementary Table 6) respectively. In the validation of external data, the accuracy, sensitivity, and specificity of the model CP were 78.50%, 78.07%, and 79.07% respectively. The accuracy, sensitivity, and specificity of the model CP left were 79.00%, 86.17%, and 72.70% respectively. The accuracy, sensitivity, and specificity of the model CP right were 83.00%, 81.82%, and 84.44% respectively. The accuracy, sensitivity and specificity of the model CP both were 82.30%, 89.50%, and 72.70% respectively (Table 3).

**Table 3:** Evaluation of model quality

Models	Accuracy (%)		Sensitivity (%)		Specificity (%)		*AUC*	*p*
	CV	EDV	CV	EDV	CV	EDV		
CP	86.860	78.500	88.000	78.070	80.500	79.070	0.917	< 0.001
CP Left	82.900	79.000	83.430	86.170	79.960	72.700	0.896	< 0.001
CP Right	82.300	83.000	85.450	81.820	79.250	84.440	0.905	< 0.001
CP Both	87.700	82.300	95.540	89.500	68.130	72.700	0.936	< 0.001

*CV* leave-one-out cross-validated method, *EDV* External data validated method

## Discussion

Our study has shown that the Fisher discriminant analysis model with carotid artery imaging can be used for predicting the carotid plaque and its location information. Furthermore, the models have excellent accuracy ranging from 78 to 90% in the prediction of carotid plaque and its location. Based on the leave-one-out cross-validation and external data validation, The average accuracy, sensitivity, and specificity was 84.94%, 88.11%, and 76.96%, when the Fisher discriminant analysis models were evaluated.

For the prediction models of cardiovascular disease, the sensitivity of the coronary heart disease prediction model established by Carlo Ricciardi is only 65.4%, which can not fully screen out patients in the crowd. Therefore, the sensitivity was mainly improved, and the sensitivity of the models established was above 80 percent in this study. The higher the sensitivity of a model, the more patients can be screened, and the better the prevention effect at the population level that can be achieved [24, 25]. Based on the population-based screening model, we specifically established the plaque location models to predict the specific location of plaque, to facilitate clinicians and imaging physicians to provide technical support for the prevention and early treatment of carotid plaque in the population.

In our study, in the model, the variables of age, hypertension, BMI, HDL, Lp (a), and BUN were all incorporated into every model building. In the discriminant model, the coefficient of hypertension and HDL was the largest, and the contribution to the discriminant was the highest. This is consistent with the following research, the morbidity of carotid plaque is very high among hypertensive patients [26–28]. HDL is a biomarker that has been discovered and has a strong guiding value in the prediction of carotid plaque [29] (Fig. 2).

Different demographic characteristics and blood biochemical indexes were used to construct models for the carotid plaque with presence, on both, the left and right sides. Although there were cross-repeated variables, the contributions of variables to model discrimination were different. Based on the results, the most important factors affecting the incidence of CP were age, hypertension, alcohol, smoking, BMI, HDL, LDL, Lp (a), GLU, BUN, UA, and APO a. However, different from our expected results, the model was not interested in blood biochemical indicators when variables were gradually included in the model, but included a lot of demographic characteristics to help distinguish them. Therefore, factors such as living habits should be noted for carotid plaque. Similarly, previous studies introduced numerous factors affecting the disease and the progress of carotid plaque. These factors were divided into six general groups: environmental factors, daily habits, risk factors, underlying diseases, mental -personality factors, and social factors [28]. Common risk factors associated with CP include hypertension, lifestyle [29], diabetes [30], obesity [31], and smoking [32]. The included variables were consistent with them, indicating the stability and reliability of the study.

In our study, there are some unique variables in the model construction. In the model CP and the model CP left, APO a and UA are unique variables. Firstly, Apolipoprotein A is known to be a protective factor of atherosclerosis, participating in lipid metabolism and preventing atherosclerosis [33]. So apolipoprotein A has a unique discriminant. And on the left side the common carotid artery (CCA) arises directly from the aortic arc, whereas on the right side the CCA originates from the innominate artery [34, 35]. UA can reach the left common carotid artery directly through the aortic arch, so UA has a discriminant effect in the left discriminant model.

There were a few potential weaknesses in the current study. Firstly, the single-site design used only provided a limited generalization of results. Secondly, to further modify and improve the prediction model, diverse statistical methods, such as artificial neural networks, decision trees or logistic regression, are worthy of being performed.

## Conclusion

Demographic characteristics and blood biochemical indexes were used, and the carotid plaque screening model was established based on Fisher discriminant analysis. On this basis, we established models to predict the location of plaque. In the models, hypertension and high-density lipoprotein made the highest contribution to the discriminant models and had the greatest influence. More attention should be paid to these two factors in the prevention of carotid plaque. And the screening models has high sensitivity and it is simple to operate in community screening. which can save resources for chronic disease prevention and to provide technical support in screening patients for doctor.

## Supplementary Information

**Additional file 1: Supplementary Table 1**. Demographic and blood biochemical indicators of external data.

**Additional file 2: Supplementary Table 2**. Collinearity diagnosis of blood biochemical indexes.

**Additional file 3: Supplementary Table 3**. Coordinates of ROC curve for the single continuous variables and FDA scores to diagnose CP.

**Additional file 4: Supplementary Table 4**. Coordinates of ROC curve for the single continuous variables and FDA scores to predict CP Left.

**Additional file 5: Supplementary Table 5**. Coordinates of ROC curve for the single continuous variables and FDA scores to predict CP Right.

**Additional file 6: Supplementary Table 6**. Coordinates of ROC curve for the single continuous variables and FDA scores to predict CP Both.

## Data Availability

The data of participants are collected by the authors and uploaded to the database, which makes it easier for the authors to use the data in the process of analyzing data and writing manuscripts. This kind of database system can conveniently shield the data irrelevant to the experiment and effectively protect the privacy of participants. The data that support the findings of this study are available from the Maanshan People's Hospital, Maanshan, China. But restrictions apply to the availability of these data, which were used under license for the current study, and so are not publicly available. Data are however available from the authors upon reasonable request and with permission of Maanshan People's Hospital, Maanshan, China. If someone wants to request the data from this study, they can contact Yufeng Wen (corresponding author).

## Abbreviations

CVD: Cardiovascular diseases
BMI: Body mass index
FDA: Fisher discrimination analysis
TC: Total cholesterol
TG: Triglycerides
GLU: Glucose
HDL: High-density lipoprotein
LDL: Low-density lipoprotein
BUN: Blood urea nitrogen
UA: Uric acid
APO a: Apolipoprotein a
APO b: Apolipoprotein b
WHO: World Health Organization
AUC: Area under the receiver operating characteristic curve
VIF: Variance inflation factor
ROC curve: Receiver operator characteristic curve
Cr: Creatinine
Lp (a): Lipoprotein (a)
CysC: Cystatin C
IMT: Intima-media thickness
