# Supplementary material for: Prediction of carotid plaque by blood biochemical indices and related factors based on Fisher discriminant analysis
Source: BMC Cardiovasc Disord. 2022 Aug 15;22:371. doi: 10.1186/s12872-022-02806-3 (PMC9377085; doi:10.1186/s12872-022-02806-3)
Supplement: Supplementary file 1 — Additional file 1: Supplementary Table 1. Demographic and blood biochemical indicators of external data. [file 12872_2022_2806_MOESM1_ESM.docx]

**supplementary Table 1** Demographic and blood biochemical indicators of external data

| Variables | Model CP  （n=200） | Model CP Left  （n=100） | Model CP Right  (n=100) | Model CP Both  (n=200) | *F/χ^2^* | *P* |
| --- | --- | --- | --- | --- | --- | --- |
| Age | 67.74±8.80 | 68.63±7.59 | 66.94±8.33 | 68.66±8.18 | 0.801 | 0.449 |
| Gender,  male(%) | 80（40.0） | 50（50.0） | 32(32.0) | 88（44.0） | 10.210 | 0.012 |
| Hypertension(%) | 145（72.5） | 69（69.0） | 66(66.0) | 150（75.0） | 32.120 | <0.001 |
| Smoking(%) | 60（30.0） | 28（28.0） | 19（19.0） | 58（29.0） | 4.356 | <0.05 |
| Alcohol(%) | 56（28.0） | 23（23.0） | 17（17.0） | 55（27.5） | 7.332 | <0.05 |
| diabetes mellitus | 95（47.5） | 32（32.0） | 29（29.0） | 91（45.5） | 6.132 | <0.001 |
| BMI | 23.56±3.28 | 23.42±3.27 | 23.1±3.23 | 24.11±3.15 | 1.690 | 0.186 |
| TC | 4.29±1.08 | 4.18±1.16 | 4.24±1.02 | 4.30±1.05 | 0.221 | 0.802 |
| TG | 1.41±0.76 | 1.49±1.06 | 1.32±0.77 | 1.48±0.85 | 0.667 | 0.517 |
| HDL | 1.18±0.30 | 1.17±0.29 | 1.23±0.35 | 1.19±0.29 | 0.527 | 0.591 |
| LDL | 2.43±0.77 | 2.29±0.83 | 2.38±0.77 | 2.39±0.79 | 0.470 | 0.625 |
| APO a | 1.21±0.20 | 1.20±0.17 | 1.22±0.23 | 1.23±0.18 | 0.865 | 0.411 |
| APO b | 0.90±0.26 | 0.89±0.30 | 0.85±0.23 | 0.89±0.27^b^ | 0.456 | 0.336 |
| Lp (a) | 216.44±150.21^b^ | 200.84±129.90 | 186.2±128.62 | 212.85±161.37^ab^ | 0.740 | 0.478 |
| GLU | 6.83±3.13^b^ | 6.44±3.03 | 5.77±2.33 | 6.65±3.01^ab^ | 2.017 | 0.134 |
| Cr | 64.43±13.79^a^ | 78.81±20.11 | 69.96±16.72 | 66.71±14.28^b^ | 13.365 | <0.001 |
| BUN | 3.69±0.51 | 5.17±1.82 | 5.71±1.81 | 3.91±0.55^ab^ | 65.296 | <0.001 |
| Cysc | 1.03±0.60 | 1.18±0.28 | 1.09±0.21 | 1.05±0.21^ab^ | 2.351 | 0.100 |
| UA | 291.74±79.36 | 329.78±90.75 | 298.65±122.52 | 297.12±78.55^ab^ | 31.419 | <0.001 |

Note: the letter a indicates comparison with the model CP left group, p< 0.05; the letter b indicates comparison with model CP right group, p< 0.05; the letter c indicates comparison with both group, p< 0.05.
