# Supplementary material for: Prediction of carotid plaque by blood biochemical indices and related factors based on Fisher discriminant analysis
Source: BMC Cardiovasc Disord. 2022 Aug 15;22:371. doi: 10.1186/s12872-022-02806-3 (PMC9377085; doi:10.1186/s12872-022-02806-3)
Supplement: Supplementary file 2 — Additional file 2: Supplementary Table 2. Collinearity diagnosis of blood biochemical indexes. [file 12872_2022_2806_MOESM2_ESM.doc]

**supplementary Table 2** Collinearity diagnosis of blood biochemical indexes

| Variables | β | S.E. | *t* | *p* | tolerance | VIF |
| --- | --- | --- | --- | --- | --- | --- |
| Constant | 0.776 | 0.066 | 11.694 | <0.001 |  |  |
| TC | 0.003 | 0.014 | 0.245 | 0.806 | 0.239 | 4.187 |
| TG | -0.03 | 0.007 | -4.040 | <0.001 | 0.611 | 1.636 |
| HDL | -0.116 | 0.039 | -2.961 | 0.003 | 0.419 | 2.384 |
| LDL | -0.05 | 0.015 | -3.352 | 0.001 | 0.339 | 2.95 |
| APO a | -0.047 | 0.058 | -0.804 | 0.421 | 0.45 | 2.22 |
| APO b | 0.061 | 0.059 | 1.032 | 0.302 | 0.245 | 4.074 |
| LP a | 0.001 | 0.001 | 10.795 | <0.001 | 0.932 | 1.073 |
| GLU | -0.001 | 0.002 | -0.333 | 0.739 | 0.892 | 1.121 |
| Cr | -0.002 | 0.001 | -6.320 | <0.001 | 0.277 | 3.611 |
| BUN | 0.005 | 0.003 | 1.566 | 0.118 | 0.648 | 1.544 |
| Cysc | 0.165 | 0.022 | 7.361 | <0.001 | 0.305 | 3.281 |
| UA | 0.001 | 0.001 | 4.129 | <0.001 | 0.821 | 1.218 |
