# Supplementary material for: Prediction of carotid plaque by blood biochemical indices and related factors based on Fisher discriminant analysis
Source: BMC Cardiovasc Disord. 2022 Aug 15;22:371. doi: 10.1186/s12872-022-02806-3 (PMC9377085; doi:10.1186/s12872-022-02806-3)
Supplement: Supplementary file 3 — Additional file 3: Supplementary Table 3. Coordinates of ROC curve for the single continuous variables and FDA scores to diagnose CP. [file 12872_2022_2806_MOESM3_ESM.docx]

**supplementary Table 3** Coordinates of ROC curve for the single continuous variables and FDA scores to diagnose CP

| Variables | Sensitivity | Specificity | AUC | *95%CI* | *P* |
| --- | --- | --- | --- | --- | --- |
| LP(a) | 0.696 | 0.589 | 0.685 | 0.660-0.710 | <0.001 |
| HDL | 0.776 | 0.377 | 0.573 | 0.546-0.600 | <0.001 |
| BMI | 0.723 | 0.422 | 0.586 | 0.560-0.611 | <0.001 |
| FDA score | 0.880 | 0.805 | 0.917 | 0.903-0.931 | <0.001 |
