# Supplementary material for: Prediction of carotid plaque by blood biochemical indices and related factors based on Fisher discriminant analysis
Source: BMC Cardiovasc Disord. 2022 Aug 15;22:371. doi: 10.1186/s12872-022-02806-3 (PMC9377085; doi:10.1186/s12872-022-02806-3)
Supplement: Supplementary file 4 — Additional file 4: Supplementary Table 4. Coordinates of ROC curve for the single continuous variables and FDA scores to predict CP Left. [file 12872_2022_2806_MOESM4_ESM.docx]

**supplementary Table 4** Coordinates of ROC curve for the single continuous variables and FDA scores to predict CP Left

| Variables | Sensitivity | Specificity | AUC | *95%CI* | *P* |
| --- | --- | --- | --- | --- | --- |
| UA | 0.657 | 0.530 | 0.581 | 0.549-0.613 | <0.001 |
| BMI | 0.631 | 0.519 | 0.589 | 0.557-0.620 | <0.001 |
| BUN | 0.595 | 0.528 | 0.534 | 0.502-0.567 | 0.036 |
| GLU | 0.790 | 0.347 | 0.504 | 0.471-0.537 | 0.807 |
| LP(a) | 0.705 | 0.587 | 0.672 | 0.642-0.702 | <0.001 |
| HDL | 0.791 | 0.377 | 0.575 | 0.543-0.607 | <0.001 |
| FDA score | 0.834 | 0.796 | 0.896 | 0.878-0.914 | <0.001 |
