# Supplementary material for: Prediction of carotid plaque by blood biochemical indices and related factors based on Fisher discriminant analysis
Source: BMC Cardiovasc Disord. 2022 Aug 15;22:371. doi: 10.1186/s12872-022-02806-3 (PMC9377085; doi:10.1186/s12872-022-02806-3)
Supplement: Supplementary file 5 — Additional file 5: Supplementary Table 5. Coordinates of ROC curve for the single continuous variables and FDA scores to predict CP Right. [file 12872_2022_2806_MOESM5_ESM.docx]

**supplementary Table 5** Coordinates of ROC curve for the single continuous variables and FDA scores to predict CP Right

| Variables | Sensitivity | Specificity | AUC | *95%CI* | *P* |
| --- | --- | --- | --- | --- | --- |
| BMI | 0.536 | 0.592 | 0.581 | 0.548-0.615 | <0.001 |
| BUN | 0.611 | 0.530 | 0.550 | 0.517-0.584 | 0.004 |
| LP(a) | 0.687 | 0.571 | 0.665 | 0.633-0.696 | <0.001 |
| LDL | 0.596 | 0.546 | 0.578 | 0.545-0.612 | <0.001 |
| HDL | 0.596 | 0.510 | 0.550 | 0.517-0.584 | 0.004 |
| FDA score | 0.855 | 0.793 | 0.905 | 0.888-0.923 | <0.001 |
