# Supplementary material for: Prediction of carotid plaque by blood biochemical indices and related factors based on Fisher discriminant analysis
Source: BMC Cardiovasc Disord. 2022 Aug 15;22:371. doi: 10.1186/s12872-022-02806-3 (PMC9377085; doi:10.1186/s12872-022-02806-3)
Supplement: Supplementary file 6 — Additional file 6: Supplementary Table 6. Coordinates of ROC curve for the single continuous variables and FDA scores to predict CP Both. [file 12872_2022_2806_MOESM6_ESM.docx]

**supplementary Table 6** Coordinates of ROC curve for the single continuous variables and FDA scores to predict CP Both

| Variables | Sensitivity | Specificity | AUC | *95%CI* | *P* |
| --- | --- | --- | --- | --- | --- |
| HDL | 0.785 | 0.377 | 0.579 | 0.551-0.607 | <0.001 |
| LP(a) | 0.860 | 0.567 | 0.695 | 0.670-0.721 | <0.001 |
| GLU | 0.781 | 0.372 | 0.503 | 0.473-0.533 | 0.814 |
| BMI | 0.729 | 0.422 | 0.586 | 0.559-0.613 | <0.001 |
| FDA score | 0.954 | 0.681 | 0.936 | 0.923-0.948 | <0.001 |
